# A knowledge synthesis of patient and public involvement in clinical practice guidelines: study protocol

**DOI:** 10.1186/1748-5908-4-30

**Published:** 2009-06-04

**Authors:** France Légaré, Antoine Boivin, Trudy van der Weijden, Christine Packenham, Sylvie Tapp, Jako Burgers

**Affiliations:** 1 Canada Research Chair in Implementation of Shared Decision Making in Primary Care, Université Laval, Quebec city, Quebec, Canada; 2 Scientific Institute for Quality of Healthcare, Radboud University Nijmegen Medical Centre, Nijmegen, the Netherlands; 3 Department of General Practice, School for Public Health and Primary Care (Caphri), Maastricht University, Maastricht, the Netherlands; 4 Ministère de la santé et des Services Sociaux de Québec, Montréal, Québec, Canada

## Abstract

**Background:**

Failure to reconcile patient preferences and values as well as social norms with clinical practice guidelines (CPGs) recommendations may hamper their implementation in clinical practice. However, little is known about patients and public involvement programs (PPIP) in CPGs development and implementation. This study aims at identifying what it is about PPIP that works, in which contexts are PPIP most likely to be effective, and how are PPIP assumed to lead to better CPGs development and implementation.

**Methods and design:**

A knowledge synthesis will be conducted in four phases. In phase one, literature on PPIP in CPGs development will be searched through bibliographic databases. A call for bibliographic references and unpublished reports will also be sent via the mailing lists of relevant organizations. Eligible publications will include original qualitative, quantitative, or mixed methods study designs reporting on a PPIP pertaining to CPGs development or implementation. They will also include documents produced by CPGs organizations to describe their PPIP. In phase two, grounded in the program's logic model, two independent reviewers will extract data to collect information on the principal components and activities of PPIP, the resources needed, the contexts in which PPIP were developed and tested, and the assumptions underlying PPIP. Quality assessment will be made for all retained publications. Our literature search will be complemented with interviews of key informants drawn from of a purposive sample of CPGs developers and patient/public representatives. In phase three, we will synthesize evidence from both the publications and interviews data using template content analysis to organize the identified components in a meaningful framework of PPIP theories. During a face-to-face workshop, findings will be validated with different stakeholder and a final toolkit for CPGs developers will be refined.

**Discussion:**

The proposed research project will be among the first to explore the PPIP in CPGs development and implementation based on a wide range of publications and key informants interviews. It is anticipated that the results generated by the proposed study will significantly contribute to the improvement of the reconciliation of CPGs with patient preferences and values as well as with social norms.

## Background

### The challenge of clinical practice guidelines (CPGs) implementation

Clinical practice guidelines (CPGs) are described as 'systematically developed statements to assist practitioner and patient decisions about appropriate health care for specific clinical circumstances'[1]. Within the knowledge to action framework, CPGs are understood as the product of a knowledge tailoring strategy, translating primary and secondary research into specific recommendations for action [2]. Their application in clinical practice is expected to improve patient outcomes by promoting an effective, equitable, and rational utilization of resources [3]. However, despite the vast amount of resources invested in CPGs development, their implementation in clinical practice remains a major challenge [4]. As a result, appropriate evidence-based care is not offered to patients, while unnecessary or harmful care often is [5-9]. An important barrier to the implementation of CPGs recommendations is their inability to reconcile patient preferences and values as well as social norms [10,11]. CPGs have also been criticized for not being responsive to increased demands from patients to share decisions with health professionals and play an active role in their care [12-14]. Furthermore, current CPGs are leaving unaddressed some of the critical challenges posed by the rising burden of chronic disease and its impact on the context of decision-making. Therefore, the role that patients and public involvement programs (PPIP) could play in CPGs development and implementation is increasingly attracting the attention of policymakers, health professionals, patients, and the public.

### The grey zone of decision making

Clinical decisions largely occur in contexts of scientific uncertainty. These grey zone (or preference sensitive) decisions are characterized either by scientific evidence that points to a balance between harms and benefits within or between options, or by the absence or insufficiency of scientific evidence [15-17]. Moreover, probabilities of risks and benefits in a population cannot be directly attributed at the individual level. Consequently, both clinicians and patients need help in resolving uncertainty when facing clinical decisions [18]. However, current CPGs are insufficiently adapted to grey zone decisions, and thus cannot help providers and their patients make informed decisions in these highly prevalent decision-making contexts.

CPGs are still largely conceived as tools that should foster adherence to a best decision defined by the 'expert health professional', rather than instruments that should support the best decision for a specific patient in a specific context. Health professionals have criticized CPGs for lacking relevant information to assist shared decision making with patients [12,19]. In Canada, a large proportion of CPGs development is undertaken by expert panels and, most of the time, patient and public organizations have a limited role to play or are at best asked to comment on draft versions of CPGs [20,21]. This is surprising because evidence suggests that patient involvement might be beneficial at different levels of health care. At the clinical level, it is associated with the quality of the decision-making process [22], reduction in unwarranted surgical interventions [23], and patients' quality of life at three years [24]. At the level of the population, patient involvement fostered by patient decision aids has been found to reduce overuse of options not clearly associated with benefits for all (e.g., prostate cancer screening) [25] and to enhance use of options clearly associated with benefits for the vast majority (e.g., cardiovascular risk factor management) [26]. The most recent systematic review of the effectiveness of patient involvement in decision making (or shared decision making) found this approach to be particularly effective in fostering adherence to the treatment choice that was made in the context of chronic disease, more specifically in the context of mental health diseases [27]. Thus, engaging patients as decision-makers, experts, and co-producers of health is particularly important in this context, as productive interactions between active and informed patients and health care providers are understood as key components to effective chronic disease management [28,29]. As decision-makers in Canada are increasingly focusing their efforts to tackle the rise of chronic diseases, the relevance for involving patients in CPGs development is thus becoming more pressing.

Beyond its role in assisting individual clinical decisions, CPGs have also a broader impact on health policy, funding decisions, and service organization [30,31]. However, social norms and economic judgments are largely implicit and poorly articulated in current CPGs, which lead to potential conflicts of interests, contradictions in CPGs recommendations, and confusion among health professionals, patients, and the public [12,32-34]. For example, the Canadian Diabetes Association recommended in 2003 that insulin glargine could be used as an alternative to generic long-acting insulin for the treatment of diabetes [20]. After reviewing virtually the same evidence, the Common Drug Review, a national advisory panel, recommended that the drug not be listed in provincial formularies on the basis of questionable added clinical benefit and a five-fold increase in price [21]. Such controversies illustrate the grey zones of decision making and the importance that CPGs developers be accountable not only to patients but also to the general public, which implies to consider cost effectiveness and cost impact [33,35-37]. The McDonnell Norms Group suggests that response to public demand and social norms be regarded as a key ingredient for the successful implementation of research evidence in clinical practice [38]. Considering the perspectives of patients and members of the public is thus a logical approach for conceptualizing the development and effective implementation of CPGs.

### International consensus on the importance of patient and public involvement in CPGs

International experience of patient and public involvement in CPGs has been accumulating in the past ten years [39]. For example, the British National Institute for Health and Clinical Excellence (NICE) has adopted a comprehensive approach to involving patients and the public in all stages of CPGs development, from the scope of CPGs topics to patient representation on CPGs development group [40]. A citizen council also ensures that members of the public can openly and transparently debate CPGs social and economic value judgments [41]. The Dutch Institute for Healthcare Improvement (CBO) has also innovated by producing patient decision aids to support grey zone decisions in existing CPGs (e.g., prostate cancer screening) [42]. In 2007, the Guideline International Network (GIN), an international network of 85 CPGs organizations, announced the creation of the GIN Patient and Public Involvement working group, thus reflecting the increasing recognition of this issue among CPGs developers [43]. In light of these initiatives, major organizations in Canada have started to call for a CPGs development process that will engage patients and the public in a more meaningful and effective way. The Canadian Medical Association, in its 2007 handbook on clinical practice guidelines, notes that patient and public involvement is 'increasingly common (and desirable) to gain input from non-health professionals and groups who are affected by the CPGs' [44]. In 2008, inspired by the British NICE, the Quebec government announced the creation of a single provincial organization that would oversee the development of all CPGs in the province to foster a more transparent and accessible platform for public and patient involvement throughout the CPGs development process [45]. Such developments could spearhead the development of structured PPIP among Canadian and international CPGs organizations, as long as decision-makers are equipped with practical knowledge to support those initiatives.

### What knowledge gaps does this study address?

Despite this growth in interest and experience, previous knowledge syntheses have left decision-makers with little practical guidance on the design of effective PPIP in CPGs development. Two recent reviews produced for the World Health Organisation (WHO) and the Cochrane collaboration found no comparative intervention study of PPIP in CPGs [46,47]. These findings indicate that the development and evaluation of PPIP are still in an early stage, and that guidance is needed to strengthen PPIP theory and effective development. However, by simply asking 'what works' and restricting their synthesis to comparative intervention studies, these reviews do not allow CPGs developers to build on the experience of other organizations and identify where efforts should be put in priority to develop effective PPIP. Furthermore, these syntheses used approaches that account neither for the high level of complexity of PPIP, the competing rationales that underpin those interventions, nor for the contextual factors that promote or impede success. Research efforts in the field of patient and public involvement must therefore move into the development of effective PPIP by focusing on more encompassing research questions [48]. Consequently, the overarching goal of this study is to strengthen the knowledge base that will support the elaboration of effective PPIP in CPGs development and implementation by undertaking a knowledge synthesis of the literature that will explore not only what works but also, how and in which context effective PPIP are developed. This in turn has the potential to foster better implementation of CPGs in clinical practice, a key need of the decision-maker partners.

### Conceptual underpinnings of this knowledge synthesis

**Figure 1:**
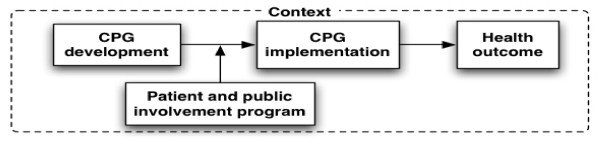
**Conceptual framework: Patients and public involvement programs in clinical practice guidelines development and implementation**.

We conceptualize a patient and public involvement program as an intervention that influences CPGs development and, indirectly, its implementation in clinical practice and health outcomes (Figure 1). Grounded in the logic model, our framework recognizes that PPIP contain a set of activities that are put forward in order to answer the needs of clients in relationship with expected outcomes [49]. In turn, these activities require specific resources (*e.g.*, human and material). Furthermore, our framework recognizes that the design and effectiveness of PPIP is influenced by the context in which they are developed.

### Research questions

This knowledge synthesis aims at identifying and refining the underlying PPIP theories by conducting a systematic literature review inspired by 'realist' methods [50]. Realist inquiries are based on a generative model of potential causality where outcome is linked to the assumed underlying mechanisms of the intervention, implemented within a specific context that will provide answers to the following research questions:

1. WHAT are the principal components and activities of PPIP that have been used to date in CPGs development? Who is involved, how are they involved, at what stage of CPGs development, and for what purpose? Which components of PPIP are perceived as important and/or effective in improving CPGs development, implementation, and/or health outcomes? What types of resources are needed to run the PPIP?

2. IN WHICH CONTEXTS have PPIP been developed and tested? What are the individual, interpersonal, institutional, and social contexts in which PPIP appear to be most effective? What factors are perceived as barriers and facilitators for the development and implementation of effective PPIP?

3. HOW are PPIP assumed to improve CPGs development, implementation, and/or quality of health care? What are the expected outcomes?

We argue that PPIPs rest on a set of expectations and assumptions that are held by their sponsors, participants, and those who judge their effectiveness [51]. These expectations constitute the underlying theory of PPIP, which provides a model of how PPIP are assumed to work [52]. PPIP theory logically links together PPIP methods, context, and outcome in a hypothesis chain, whose generic format is: 'if a specific patient and public involvement program is implemented within a given context, it will then impact on the CPGs development process, implementation, and/or health outcome.' In other words, this knowledge synthesis will take into account context as an essential element for improving our understanding of PPIP in CPGs development and implementation.

## Methods and design

The proposed knowledge synthesis is comprised of four main phases.

### Phase one: Search for evidence

#### Search strategy

With the help of an information specialist, English and French publications up to January 2009 will be identified through: bibliographic databases (*e.g.*, Cochrane Consumers and Communication Review Group's Specialized Register, the Cochrane Controlled Trials Register, MEDLINE, EMBASE, CINAHL, PsycINFO, Sociological Abstracts, G-I-N database) [53]; manual search of key journals and of the G-I-N conference proceedings; personal contact with key authors and experts in CPGs development using the network of G-I-N; and reference lists of included studies and systematic reviews. A call for bibliographic references and unpublished reports will also be sent via the mailing lists of the G-I-N Patient and Public Involvement Working Group. Our decision-maker partners will be consulted to help in this search for evidence. A list of publications considered eligible by the research team will be used to devise the search strategy and compute the precision of our search [54].

### Inclusion and exclusion criteria

#### Types of studies

Eligible publications will include original qualitative, quantitative or mixed methods study designs (*i.e.*, case study, observational, and intervention studies). They will also include documents produced by national/governmental supported/non-profit CPGs organizations to describe their PPIP. Studies focused on PPIP in other areas of health care (*e.g.*, health technology assessment, health research, planning and delivery of health services, development of health information material) will be excluded. One team member is currently involved in two other knowledge syntheses that share a similar focus. One deals with patients' perspective on electronic health record [55], the other deals with patients and public involvement in health technology assessment [56]. Also, another team member is involved with the International Patient Decision Aids Standards (IPDAS) Collaboration, a group dedicated to patients' involvement in healthcare decisions [57].

#### Participants

Patients refer to people with personal experience of the disease, health interventions or services discussed in CPGs (including family members and carers). The public refers to members of society interested in health care services and whose life may be affected directly or indirectly by a specific CPG [58].

#### Intervention

PPIP refers, at the minimum, to one formal method of involving patients and/or the public in CPGs development. Formal involvement methods may include: communication (information is communicated to patients or the public); consultation (information is collected from patients or the public); or participation (patients or the public participate in an exchange of information and deliberation with other CPGs developers) [59]. CPGs development is defined as the systematic process leading to the production of statements to assist practitioner and patient decisions about appropriate health care for specific clinical circumstances [1]. Our definition of CPGs development is purposefully broad as to include CPGs implementation strategies dealing with patient-mediated interventions (*e.g.*, communication of information to patients and the public about CPGs, production of patient/public versions of CPGs and the integration of patient decision aids in existing CPGs). We excluded other CPGs implementation strategies (*e.g.*, audit and feedback, education, organizational change) because of our decision-maker partners priorities and of the practical challenge of concurrently addressing PPIP in CPGs development and all possible strategies of implementation [4,5].

### Phase two: Appraise and extract data from identified primary studies

#### Study identification and data extraction

A research assistant will screen all references. Potentially eligible references will be reviewed by the two co-PIs independently. Any discrepancies between the two reviewers on study inclusion will be resolved by discussion with other team members, including at least one of our decision-maker partners. All eligible references will then be extracted by pairs of research team members using a data extraction form that was developed from previous work in this field [58,60-62]. Pilot testing of the standardized form will be conducted and its results discussed by team members to finalize the form. Pairs of reviewers will compare abstracted information and disagreements will be resolved through consensus. Information will be collected on:

1. Bibliographic reference, type of publication, and study design.

2. Principal components of PPIP, including: planned activities, who is involved, how they are involved, how they are trained or guided, their level of decision-making power, at what stage of CPGs development, and for what purpose; components that seem the most important and effective; and resources needed (research question one).

3. Context in which PPIP are developed and tested, including individual, interpersonal, institutional, and social context factors; factors perceived as barriers and facilitators for the development and implementation of effective PPIP (research question two).

4. PPIP theory: explicit and implicit assumptions regarding how PPIPs are deemed to lead to improved CPGs development, implementation, and/or health outcomes (research question three) [60,63]

#### Quality assessment

Study quality will be assessed by two independent reviewers and based on two main criteria: relevance (whether the authors of the included publication are explicit about the principal components of PPIPs that have been used in CPGs development), and rigor (whether the study can make a credible contribution in terms of validity and reliability). Quality criteria developed for mixed methods review will be used [64].

#### Data validation

Key informants will be drawn from a purposive sample of six to ten CPGs developers and patient/public representatives working with organizations with a PPIP. Individual phone interviews with key informants will serve as a method for complementing and validating data extraction from publications. Examples of questions in the interview guide include: descriptive information on existing PPIPs and their context of development, components of PPIPs that seem the most important and effective; perceived barriers and facilitators for the development and implementation of effective PPIPs; examples of best (and 'bad') practices. Interviews will be recorded and transcribed verbatim. The appropriate software will be used for qualitative analyses to support data collection, organization, and analysis.

### Phase three: Synthesize evidence and draw conclusions

Both publication and interview data will be analyzed. A research assistant will enter findings into a data matrix to facilitate comparison of how each publication performs on principal components of each PPIP. For each publication and interview, template content analysis will be used to organize its identified set of principal components into a meaningful framework of PPIP theories [65]. Thus, based on a taxonomy of PPIP theories, we will identify and classify existing PPIP theories based on the principal components that will have been extracted from each study. This taxonomy was previously developed by one of the author based on qualitative interviews with CPGs developers [14]. For example, the 'health care governance' PPIP theory holds that consultation with a statistically representative group of patients in the summary of evidence stage of CPGs development should result in improved patient adherence with cost-effective interventions. In the context of this synthesis, the taxonomy of PPIP theories will be refined and expanded to include contextual factors that are seen as influencing PPIP effectiveness.

### Phase four: Achieve consensus with our decision-maker partners on a proposed toolkit on PPIP that could be tested in a subsequent study

In consultation with our decision-maker partners, we will engage in a consensus process for developing a toolkit on effective PPIP in CPGs development that could be tested in a subsequent study with the potential target users. We will use the PPIP theories resulting from this knowledge synthesis as background evidence to inform an international consensus on best practices in PPIP. In line with our concern with contextual factors, we will not aim at developing a monolithic set of recommendations on 'what works' but rather provide decision-makers with a toolkit of key issues to consider when designing, implementing, and evaluating PPIP in specific contexts of CPGs development. The consensus process will involve: the production of a background evidence document and draft quality criteria based on the knowledge synthesis; recruitment of participant stakeholder groups (including patient/public representatives, CPGs developers, health professionals, and government representatives); and refinement of the toolkit in a face-to-face workshop held at one of the stakeholders' conference meeting. Topics addressed in the workshop will include: reaction of participants to the findings from the knowledge synthesis, proposed changes to the toolkit, barriers and facilitators to implementing this toolkit in CPGs development, and recommendations for future research. We will also collect information on the demographic characteristics of the participants and additional information on their organizations.

### Strategies to ensure methodological rigor

To minimize bias, a standard checklist of inclusion/exclusion criteria and a data extraction sheet will be piloted and refined by two team members. One reviewer will apply the inclusion/exclusion criteria to the result of the searches. Two reviewers will independently perform data extraction, classification, and analysis of the included studies and interviews. Any contentious results will be referred to the research team. With the aim of verifying credibility of the findings, a summary of the data extraction of the identified publication will be sent to the concerned authors (member checking) [66] who will be invited to make additional comments or corrections. A log book and audit trail will be kept and be made available for an external assessor. Findings and recommendations from the review will be validated through group debriefing within the research team and research advisory committee during the synthesis, and our consensus procedure with CPGs developers and patient/public organizations to develop final recommendations.

### Ethical considerations

All documents collected for the knowledge synthesis will be obtained from publicly available sources. Participants in the individual interviews will be asked to complete a consent form presenting research objectives and information about research implications. Participants to the Delphi web-based exercise study will be informed that they consent to participate when creating their electronic account. Ethics approval for the project has been received from the Research Ethics Board of the Centre Hospitalier Universitaire de Québec (approved 18 December 2008; ethics number 5-08-12-07).

## Discussion

The main decision-makers and stakeholders of this knowledge synthesis are patients, public, government, and health professional organizations in Canada and abroad that are interested in, or affected by, CPGs development. Knowledge translation researchers will also be interested in our results given their potential to advance a new paradigm in knowledge science: one that acknowledges the contribution of patients and the public in the creation and application of knowledge.

This knowledge synthesis will provide decision-makers with the essential knowledge that is needed for elaborating effective PPIP in CPGs development and implementation, notably through the creation of an evidence-based toolkit. CPGs developers will then better be able to understand the conditions where PPIP are likely to be most effective and which resources need to be prioritized when designing such programs. Furthermore, insights into the inner mechanisms of involvement strategies will lay the foundation for a consensus on how to involve patients and the public within specific contexts of CPGs development and implementation. Also, our research team will be in a unique position to perform a comparative analysis of patients and public involvement in a number of key areas of healthcare services and systems: electronic health records [55], health technology assessment [56], patients' decision aids [67], and CPGs, the focus of this knowledge synthesis. This proposal is directly linked with policy-making priorities at the Canadian Institute of Health Research (CIHR), the funding agency for this research initiative. Its Partnerships and Citizen Engagement Branch is committed to ensure the effective management of public engagement activities and foster research in knowledge management, values-based decision-making, and public engagement [68]. Production of the synthesis could lead to greater public legitimacy, acceptability, and effectiveness of CPGs implementation.

## Competing interests

The authors declare that they have no competing interests.

## Authors' contributions

FL and AB developed the research protocol and all authors contributed to the final version. FL is its guarantor. All authors read and approved the final manuscript.
